# Dataset on the effect of nano-modified additives of concrete mixes technological properties for winter concreting

**DOI:** 10.1016/j.dib.2020.105756

**Published:** 2020-05-27

**Authors:** Alexander P. Svintsov, Vera V. Galishnikova, Nadezhda А. Stashevskaya

**Affiliations:** Peoples' Friendship University of Russia (RUDN University), 6 Miklukho-Maklaya Street, Moscow, 117198, Russian Federation

**Keywords:** Permanent formwork, Cement-particle board, Quality, Crack

## Abstract

This article contains data of effect of nano-modified additives on the technological properties of concrete mixes for winter concreting. The nano-modified additive, consisting of naphthalene formaldehyde, nano-modified silicon dioxide, saponified wood resin and sodium nitrate, ensures the manufacturability of the concrete mix, its quality laying and normal curing at low outdoor temperatures. The nano-modified additive allows prevents the phenomenon of segregation of concrete mixes of grade C12/15. The application of nano-modified additive together with sodium nitrate (4 wt.% of cement) ensures normal conditions for the hydration of cement paste at ambient temperatures from +5 °C to –5 °C. This dataset is associated with a research article entitled “Effect of nano-modified additives on properties of concrete mixtures during winter season” [1].

Specifications table

**Table:** 

**Subject**	Civil and Structural Engineering
**Specific subject area**	Concrete mixes for construction at low temperatures
**Type of data**	Table
**How data were acquired**	For weighing materials, scales with a weighting limit of 10 kg with the division scale of 1 g was used. For the production of concrete mix, a gravitational concrete mixer was used. Vibrating table with the adjustable frequency of 4000–10,500 rev/min, a timer and a clamping tool were used for compacting the concrete mix in the forms of cubes. To determine the compressive strength of concrete specimens, a 1500 kN hydraulic press was used. The study of the separation of the concrete mixture was carried out using Static Segregation Column Mold-HC-3666.
**Data format**	Raw Analyzed
**Parameters for data collection**	Laboratory conditions were used to collect data on the flowability and segregation of the concrete mix and the strength of the concrete for axial compression. To harden the concrete mix at low temperatures, the conditions of the construction site are used.
**Description of data collection**	Measurement of slump test of concrete mix; measurement of flowability of concrete mix; the test of concrete mix at the risk of segregation; the compressive strength of concrete specimens.
**Data source location**	Peoples' Friendship University of Russia (RUDN University), Moscow, Russian Federation
**Data accessibility**	With the article
**Related research article**	[1] Alexander P. Svintsov, Evgeny L. Shchesnyak, Vera V. Galishnikova, Roman S. Fediuk, Nadezhda А. Stashevskaya. Effect of nano-modified additives on properties of concrete mixtures during winter season, Constr. Build. Mat. 237 (2020) 117,527. https://doi.org/10.1016/j.conbuildmat.2019.117527

## Value of the data

• These data have a practical importance for ensuring the hydration of Portland cement under ambient conditions of temperature up to –5 °С.
• This data can be useful for concrete plants that are engaged in concrete work in the cold season.
• These data can be used together with other sets of similar data to develop a study of the effect of additives on the properties of concrete mixes.
• This data can be used to solve engineering problems of improving the technology of construction of reinforced concrete building structures.
• This data can be used for comparison with data on the influence of other types of additives on the properties of concrete mixes.
• These data serve as the basis for identifying the cause-effect relationships of nano-modified additives on the technological features of concrete mixes and the strength properties of the obtained concrete.

## Data description

The presented materials are used for the preparation of concrete mixes for concrete grade C12/15 (Table 1). To solve the technological problems of concreting in cold weather, the additives have been taken, which allow to obtain concrete mixes with desired properties: sulfo-naphthalene-formaldehyde based superplasticizer (SNF), wood saponified resin (WSR), antifreeze additive of sodium nitrate (NaNO_3_), nanosilica (nano-SiO_2_). The determination of the properties of concrete mixes was performed with a constant volume of water for mixing and a variable amount of Portland cement. This allowed to create conditions of equal initial flowability of the concrete mix without introducing a nano-modified superplasticizer. Superplasticizer contributes to increase the setting time. In terms of winter concreting, this is a negative property, since it requires the shortest possible time for setting and strength development. To neutralize the influence of the said factor on the concrete mix, it is of interest to use a superplasticizer of naphthalene formaldehyde, nanomodified with silicon dioxide in the ratio of (SNF+nano-SiO_2_)=(0.2 to 1.0)+0.1. The main indicator of the effectiveness of superplasticizers on concrete mix is the degree of their liquefaction ability. The mobility of the concrete mix varies depending on the amount of added nanosuperplasticizer. A mix without additives with slump of 2–6 cm was accepted as a reference standard. Increasing the flowability of concrete mix is associated with a risk of its segregation. This is especially typical for concretes C12/15. Determination of the segregation resistance of the concrete mix was carried out according to the criterion of solution separation. Table 2 presents the data on the changes in the flowability and segregation of the concrete mix depending on the amount of added nano-modified superplasticizer (SNF+nano-SiO_2_) and water-cement ratio (W/C). To reduce the risk of segregation of the components of concrete mix, a nano-modified superplasticizer with air-entraining additive (AEA) is proposed (SNF+nano-SiO_2_)=(0.2 to 1.0)+0.1. As an air-entraining additive, saponified wood resin (WSR) was adopted in the dosages (%) of 0.01; 0.05; 0.07. The separate introduction of the air-entraining additives increases the flowability of the concrete mix. The combined introduction of the air-entraining component and the nanosuperplasticizer is a new additive composition that allows to obtain a synergistic effect of preventing segregation of the concrete mix with high flowability. Moreover, this composition has a plasticizing effect on concrete mixes with different values of w/c. Changes in flowability and segregation of the concrete mix depending on the amount of added nanosuperplasticizer (SNF+nano-SiO_2_)=(0.2 to 1.0)+0.1, air-entraining additive (WSR) in the dosages (%) of 0.01; 0.05; 0.07 and water-cement ratio (W/C) are presented in tables 3–5. The specimens were tested for compressive strength after 28 days of curing in the normal curing chamber. The minimum number of samples taken is *n* = 5. The number of specimens without modifying additives was 3 cubes for each composition. Concrete grade of C12/15 with the required compressive strength *f_ck, cube_*=19.6 MPa. Table 6 presents the data on the changes in compressive strength of concrete samples of class C12/15 depending on the amount of nanosuperplasticizer (SNF+nano-SiO_2_)=(0.2 to 1.0)+0.1 at the water-cement ratios (W/C) of 0.65; 0.56; 0.50. The application of air-entraining additives (AEA) in addition to the nano-modified superplasticizer reduces the rheological effect of segregation of components. Table 7, Table 8, Table 9 present the data on the changes in compressive strength of concrete samples depending on the amount of nanosuperplasticizer (SNF+nano-SiO_2_)=(0.2 to 1.0)+0.1 and air-entraining additive (WSR) in the dosages (%) of 0.01; 0.05; 0.07 at the water-cement ratios (W/C) of 0.65; 0.56; 0.50. A nano-modified additive was used to ensure the hydration of Portland cement in an environment with a temperature of +5 °C and below, as well as to create the required flowability of the concrete mix without segregation of the components. The additive consists of four components (wt.% of Portland cement): SNF - 0.4; WSR – 0.05; NaNO_3_ – 4.0; nano-SiO_2_ – 0.1. An experimental verification of the nano-modified concrete mix was carried out during the construction of the slab of a five-story car parking building. Control specimens were tested in the laboratory after 3, 7, 14 and 28 days of curing. To determine the strength of concrete in the slab, two cores with the diameter of 100 mm were taken. The cores were removed from the slab after 28 days of curing. Table 10 presents the data on changes in the strength of concrete samples cured in implementation conditions at a construction site. Table 11 presents the data on changes in the strength of cores extracted from the slab.

**Table 1:** Compositions of concrete mixes.

Materials	Weight for 1 m^3^		
	Mixing number		
	1	2	3
Cement 42.5 (kg)	230	270	300
Crushed granite 20 mm (kg)	550	550	525
Crushed granite 10 mm (kg)	550	550	525
Construction sand (kg)	750	700	690
Water (l)	150	150	150
Water to cement ratio (W/C)	0.65	0.56	0.50

**Table 2:** Changes in the flowability and segregation of the concrete mix depending on the amount of added nanosuperplasticizer (SNF+nano-SiO_2_) and water-cement ratio (W/C).

SNF+nano-SiO_2_ (by wt.% of cement)	W/*C* = 0.65	W/*C* = 0.56	W/*C* = 0.50
	Flowability of concrete mix		
	Slump (cm)		
0.0	2	3	3
0.20+0.1	6	8	9
0.40+0.1	10	14	17
0.60+0.1	15	18	20
0.80+0.1	16	19	22
1.00+0.1	17	20	23
		Slump flow (cm)	
0.0	21	21	22
0.20+0.1	25	30	36
0.40+0.1	36	40	41
0.60+0.1	40	44	55
0.80+0.1	46	50	58
1.00+0.1	47	52	60
	Segregation resistance (%)		
0.0	10	9	8
0.20+0.1	9	8.5	8
0.40+0.1	9	8.5	7.5
0.60+0.1	9	8	7
0.80+0.1	8	7	6
1.00+0.1	7	6	5.5

**Table 3:** Changes in flowability and segregation of the concrete mix depending on the amount of added nanosuperplasticizer (SNF+nano-SiO2), air-entraining additive (WSR) in a dosage (%) of 0.01 and water-cement ratio (W/C).

(SNF+nano-SiO_2_)+WSR (by wt.% of cement)	W/*C* = 0.65	W/*C* = 0.56	W/*C* = 0.50
	Flowability of concrete mix		
	Slump (cm)		
0.0	3.0	3.0	2.0
(0.20+0.1)+0.01	9.0	8.0	9.0
(0.40+0.1)+0.01	15.0	14.0	13.0
(0.60+0.1)+0.01	19.0	17.0	15.0
(0.80+0.1)+0.01	19.0	19.0	18,0
(1.00+0.1)+0.01	20.0	19.0	19.0
		Slump flow (cm)	
0.0	22.0	22.0	22.0
(0.20+0.1)+0.01	25.0	28.0	30,0
(0.40+0.1)+0.01	35.0	38.0	44.0
(0.60+0.1)+0.01	41.0	47.0	53.0
(0.80+0.1)+0.01	42.0	48.0	52.0
(1.00+0.1)+0.01	43.0	51.0	55.0
	Segregation resistance (%)		
0.0	8.0	9.0	8.0
(0.20+0.1)+0.01	2.5	2.0	3.0
(0.40+0.1)+0.01	2.5	2.0	2.5
(0.60+0.1)+0.01	3.0	3.0	4.0
(0.80+0.1)+0.01	4.0	2.0	4.0
(1.00+0.1)+0.01	4.0	3.0	4.0

**Table 4:** Changes in flowability and segregation of the concrete mix depending on the amount of added nanosuperplasticizer (SNF+nano-SiO2), air-entraining additive (WSR) in a dosage (%) of 0.05 and water-cement ratio (W/C).

(SNF+nano-SiO_2_)+WSR (by wt.% of cement)	W/*C* = 0.65	W/*C* = 0.56	W/*C* = 0.50
	Flowability of concrete mix		
	Slump (cm)		
0.0	3.0	3.0	2.0
(0.20+0.1)+0.05	10.0	11.0	9.0
(0.40+0.1)+0.05	16.0	15.0	13.0
(0.60+0.1)+0.05	20.0	19.0	17.0
(0.80+0.1)+0.05	22.0	21.0	20.0
(1.00+0.1)+0.05	23.0	23.0	21.0
	Slump flow (cm)		
0.0	24.0	23.0	21.0
(0.20+0.1)+0.05	30.0	28.0	28.0
(0.40+0.1)+0.05	45.0	36.0	38.0
(0.60+0.1)+0.05	44.0	44.0	36.0
(0.80+0.1)+0.05	60.0	48.0	44.0
(1.00+0.1)+0.05	58.0	52.0	46.0
	Segregation resistance (%)		
0.0	8.0	6.0	6.0
(0.20+0.1)+0.05	2.0	2.0	2.0
(0.40+0.1)+0.05	2.0	3.0	2.0
(0.60+0.1)+0.05	2.0	2.5	3.0
(0.80+0.1)+0.05	3.0	3.0	3.0
(1.00+0.1)+0.05	3.5	3.0	3.0

**Table 5:** Changes in flowability and segregation of the concrete mix depending on the amount of added nanosuperplasticizer (SNF+nano-SiO2), air-entraining additive (WSR) in a dosage (%) of 0.07 and water-cement ratio (W/C).

(SNF+nano-SiO_2_)+WSR (by wt.% of cement)	W/*C* = 0.65	W/*C* = 0.56	W/*C* = 0.50
	Flowability of concrete mix		
	Slump (cm)		
0.0	3.0	4.0	3.0
(0.20+0.1)+0.07	15.0	13.0	12.0
(0.40+0.1)+0.07	18.0	15.0	16.0
(0.60+0.1)+0.07	18.0	19.0	18.0
(0.80+0.1)+0.07	21.0	21.0	20.0
(1.00+0.1)+0.07	24.0	22.0	21.0
	Slump flow (cm)		
0.0	22.0	22.0	21.0
(0.20+0.1)+0.07	36.0	33.0	30.0
(0.40+0.1)+0.07	38.0	39.0	37.0
(0.60+0.1)+0.07	54.0	46.0	40.0
(0.80+0.1)+0.07	58.0	50.0	44,0
(1.00+0.1)+0.07	60.0	52.0	46.0
	Segregation resistance (%)		
0.0	5.0	6.0	5.0
(0.20+0.1)+0.07	1.0	3.0	1.0
(0.40+0.1)+0.07	1.0	2.0	2.0
(0.60+0.1)+0.07	2.0	2.0	2.0
(0.80+0.1)+0.07	2.0	3.0	2.0
(1.00+0.1)+0.07	2.0	3.0	2.0

**Table 6:** Changes in compressive strength of concrete samples depending on the amount of nanosuperplasticizer (SNF+nano-SiO_2_) and water-cement ratio (W/C).

SNF+nano-SiO_2_ (by wt.% of cement)	Compressive strength (MPa)					
	Number of samples					Average
	1	2	3	4	5	
	W/*C* = 0.65					
0.0	18.2	18.6	17.6	–	–	18.1
0.20+0.1	17.2	18.2	18.6	18.9	18.6	18.3
0.40+0.1	18.4	18.1	18.6	18.3	17.8	18.2
0.60+0.1	18.6	18.4	17.8	18.1	17.6	18.1
0.80+0.1	17.8	16.4	17.1	17.6	17.3	17.2
1.00+0.1	16.6	15.8	16.2	16.8	15.4	16.2
	W/*C* = 0.56					
0.0	18.4	18.2	18.9	–	–	18.5
0.20+0.1	18.0	18.2	19.1	18.6	18.4	18.5
0.40+0.1	18.4	18.2	18.5	18.8	18.6	18.5
0.60+0.1	18.5	18.7	18.2	17.8	18.7	18.4
0.80+0.1	18.6	18.4	17.8	18.0	18.5	18.3
1.00+0.1	17.8	18.0	17.6	17.2	16.2	17.4
	W/*C* = 0.50					
0.0	19.6	17.8	20.1	–	–	19.2
0.20+0.1	19.0	19.4	18.8	19.1	19.2	19.1
0.40+0.1	18.8	19.6	19.1	19.3	18.7	19.1
0.60+0.1	19.6	18.8	18.9	18.2	19.5	19.0
0.80+0.1	18.9	18.6	19.1	19.0	18.0	18.7
1.00+0.1	18.8	17.6	18.2	17.4	17.2	17.8

**Table 7:** Changes in compressive strength of concrete samples depending on the amount of nanosuperplasticizer (SNF+nano-SiO_2_), air-entraining additive (WSR) in a dosage of 0.01% and water-cement ratio (W/C).

(SNF+nano-SiO_2_)+WSR (by wt.% of cement)	Compressive strength (MPa)					
	Number of samples					Average
	1	2	3	4	5	
	W/*C* = 0.65					
0.0	18.2	18.6	17.6	–	–	18.1
(0.20+0.1)+0.01	18.6	17.7	19.1	18.3	18.2	18.4
(0.40+0.1)+0.01	18.8	18.7	19.0	17.4	18.0	18.4
(0.60+0.1)+0.01	18.6	19.1	18.9	17.6	17.8	18.4
(0.80+0.1)+0.01	18.4	18.6	17.7	17.6	18.9	18.2
(1.00+0.1)+0.01	17.9	17.6	18.0	17.5	17.9	17.8
	W/*C* = 0.56					
0.0	18.4	18.2	18.9	–	–	18.5
(0.20+0.1)+0.01	19.1	18.3	18.4	18.8	18.2	18.6
(0.40+0.1)+0.01	17.6	19.6	18.8	18.4	19.1	18.7
(0.60+0.1)+0.01	19.6	19.0	17.5	17.6	19.8	18.7
(0.80+0.1)+0.01	17.6	18.5	18.3	19.1	18.6	18.4
(1.00+0.1)+0.01	18.7	17.6	17.8	17.9	18.6	18.1
	W/*C* = 0.50					
0.0	19.6	17.8	20.1	–	–	19.2
(0.20+0.1)+0.01	19.2	19.1	19.4	19.5	18.7	19.2
(0.40+0.1)+0.01	18.9	19.5	19.7	19.6	18.9	19.3
(0.60+0.1)+0.01	19.2	19.4	19.4	19.0	19.2	19.2
(0.80+0.1)+0.01	18.9	19.3	19.4	18.6	18.2	18.9
(1.00+0.1)+0.01	18.6	19.1	18.4	17.8	18.0	18.4

**Table 8:** Changes in compressive strength of concrete samples depending on the amount of nanosuperplasticizer (SNF+nano-SiO_2_), air-entraining additive (WSR) in a dosage of 0.05% and water-cement ratio (W/C).

(SNF+nano-SiO_2_)+WSR (by wt.% of cement)	Compressive strength (MPa)					
	Number of samples					Average
	1	2	3	4	5	
	W/*C* = 0.65					
0.0	18.2	18.6	17.6	–	–	18.1
(0.20+0.1)+0.05	18.8	19.2	17.9	18.7	19.4	18.8
(0.40+0.1)+0.05	19.5	19.1	18.5	19.2	18.2	18.9
(0.60+0.1)+0.05	17.8	19.5	19.6	17.9	19.7	18.9
(0.80+0.1)+0.05	18.6	18.9	19.0	18.6	18.4	18.7
(1.00+0.1)+0.05	18.3	18.5	17.6	18.4	18.7	18.3
	W/*C* = 0.56					
0.0	18.4	18.2	18.9	–	–	18.5
(0.20+0.1)+0.05	19.8	19.4	18.4	19.6	18.8	19.2
(0.40+0.1)+0.05	18.8	19.7	19.8	19.6	18.6	19.3
(0.60+0.1)+0.05	19.2	19.3	18.8	19.8	19.4	19.3
(0.80+0.1)+0.05	19.1	19.4	19.4	18.7	18.9	19.1
(1.00+0.1)+0.05	19.1	18.6	18.7	17.9	18.7	18.6
	W/*C* = 0.50					
0.0	19.6	17.8	20.1	–	–	19.2
(0.20+0.1)+0.05	19.1	19.4	19.0	19.2	19.6	19.3
(0.40+0.1)+0.05	19.3	19.5	19.7	18.5	19.4	19.3
(0.60+0.1)+0.05	19.6	18.3	19.5	19.5	19.1	19.2
(0.80+0.1)+0.05	18.2	19.8	19.0	17.9	18.8	18.7
(1.00+0.1)+0.05	18.1	18.4	17.9	17.7	18.4	18.1

**Table 9:** Changes in compressive strength of concrete samples depending on the amount of nanosuperplasticizer (SNF+nano-SiO_2_), air-entraining additive (WSR) in a dosage of 0.07% and water-cement ratio (W/C).

(SNF+nano-SiO_2_)+WSR (by wt.% of cement)	Compressive strength (MPa)					
	Number of samples					Average
	1	2	3	4	5	
	W/*C* = 0.65					
0.0	18.2	18.6	17.6	–	–	18.1
(0.20+0.1)+0.07	17.6	18.4	18.7	18.3	18.0	18.2
(0.40+0.1)+0.07	19.1	18.6	17.0	18.8	17.9	18.3
(0.60+0.1)+0.07	17.3	17.6	19.2	18.9	18.5	18.3
(0.80+0.1)+0.07	18.5	18.7	17.1	18.4	17.8	18.1
(1.00+0.1)+0.07	18.6	16.5	17.4	17.8	17.0	17.5
	W/*C* = 0.56					
0.0	18.4	18.2	18.9	–	–	18.5
(0.20+0.1)+0.07	19.6	18.6	19.4	18.6	19.7	19.2
(0.40+0.1)+0.07	19.8	19.6	18.9	19.4	18.7	19.3
(0.60+0.1)+0.07	19.5	19.4	19.7	18.4	19.4	19.3
(0.80+0.1)+0.07	19.5	19.4	19.2	18.7	18.7	19.1
(1.00+0.1)+0.07	18.8	18.2	19.4	18.1	18.7	18.6
	W/*C* = 0.50					
0.0	19.6	17.6	20.1	–		19.1
(0.20+0.1)+0.07	19.8	19.0	19.4	19.6	18.7	19.3
(0.40+0.1)+0.07	18.9	19.8	19.6	19.2	19.0	19.3
(0.60+0.1)+0.07	19.1	18.8	19.4	19.9	19.5	19.3
(0.80+0.1)+0.07	19.0	18.0	19.8	18.9	18.5	18.8
(1.00+0.1)+0.07	17.2	18.4	17.7	18.0	16.4	17.5

**Table 10:** The compressive strength of concrete samples after curing at a construction site.

Curing period (Days)	Compressive strength (MPa)					
	Number of samples					Average
	1	2	3	4	5	
3	4.6	3.8	5.2	4.4	4.1	4.42
7	6.1	6.3	7.5	7.7	6.2	6.76
14	12.2	15.2	12.7	17.2	10.5	13.56
28	19.8	19.7	20.9	18.9	18.8	19.6

**Table 11:** Compressive strength of cores after curing in the slab.

Curing period (Days)	Compressive strength (MPa)		
	Number of samples		Average
	1	2	
28	18.6	19.4	19.0

## Experimental design, materials, and methods

For the production of concrete mix, a gravitational concrete mixer was used. The volume of the mixer drum is 0.03 m^3^, and the volume of the stirred mix is 0.02 m^3^. Mixing of materials were carried out until a homogeneous mix was obtained. The obtained concrete mix was used to determine its flowability, segregation, and production of specimens to determine the compressive strength. Cubic specimens measuring 100 × 100 × 100 mm were produced from the obtained mixes. In accordance with the plan of the experiment, the minimum number of specimens for each test was *n* = 5. The number of specimens without modifying additives was 3 cubes for each composition. The number of specimens produced in accordance with table 3 was 324 units. Concrete grade of C12/15 with the required compressive strength *f_ck, cube_*=19.6 MPa. After 24 h, concrete specimens were removed from the forms. Concrete specimens were stored in a normal curing chamber with automatic maintenance of temperature and humidity for 28 days. The flowability of the concrete mix was determined by measuring the slump and the diameter of its flow. Determination of the segregation resistance of the concrete mix was carried out according to the criterion of solution separation. The study of the separation of the concrete mixture was carried out using Static Segregation Column Mold-HC-3666. It was used to determine the potential for static separation of the mix by measuring the coarse aggregate content in the upper and lower parts of the cylindrical column. During the tests, the concrete mix was poured into the top of the column and held for 15 min. Then, coarse aggregate from the upper and lower parts of the column was collected, washed from the cement paste and sieved through a sieve with 4.75 mm mesh. The segregation index (*SI*) was calculated by comparing the masses in the upper (*M_u_*) and lower parts (*M_l_*). To determine the compressive strength of concrete specimens, a 1500 kN hydraulic press was used. The specimens were tested for compressive strength after 28 days of curing in the normal curing chamber. A nano-modified additive was used to ensure the hydration of Portland cement in an environment with a temperature of +5 °C and below, as well as to create the required flowability of the concrete mix without segregation of the components. An experimental verification of the nano-modified concrete mix was carried out during the construction of the slab of a five-story car parking building. The prepared concrete mix was laid with compaction with a surface vibrator. The surface of the concreted slab was covered with 50 mm thick mineral wool insulation. Slab and control specimens were cured in natural conditions. The average daily outside air temperature at the site of study (Moscow) in the period from 20 October 2018 to 17 November 2018 ranged from +5 °C to –6 °C. The outdoor temperature was measured twice a day with an interval of 12 h. Visual observation of the condition of slab and control specimens was performed every 24 h. Control specimens were tested in the laboratory after 3, 7, 14 and 28 days of curing. The cores were removed from the slab on 17 November 2018 after 28 days of curing.

## Declaration of Competing Interest

The authors declare that they have no known competing financial interests or personal relationships which have, or could be perceived to have, influenced the work reported in this article.

## Appendix Supplementary materials

Image, application 1
